# Physiological oxygen levels reset K^+^ channel activity in human vascular endothelial cells

**DOI:** 10.1016/j.redox.2025.103981

**Published:** 2025-12-18

**Authors:** Fan Yang, Ashia Wheeler-Crawford, Alan McIntyre, Giovanni E. Mann, Joern R. Steinert

**Affiliations:** a School of Cardiovascular and Metabolic Medicine & Sciences, King's British Heart Foundation Centre of Research Excellence, Faculty of Life Sciences & Medicine, King's College London, 150 Stamford Street, London, SE1 9NH, UK; b Centre for Cancer Sciences, Biodiscovery Institute, School of Medicine, University of Nottingham, Nottingham, NG7 2UH, UK; c School of Life Sciences, Faculty of Medicine and Health Sciences, University of Nottingham, Nottingham, NG7 2UH, UK

**Keywords:** Endothelial cells, Potassium channels, Nitric oxide, Physiological oxygen tension, Physiological normoxia, Redox signalling

## Abstract

Human endothelial cells (EC) play a critical role in vascular homeostasis and their function is influenced by oxygen tension. This study investigates for the first time the effects of long-term adaptation (5 days) of two major EC types to physiological oxygen tension (5 kPa) on basal and nitric oxide (NO)-modulated K^+^ channel activities. Whole-cell patch clamp experiments demonstrate that human umbilical vein EC (HUVEC) exhibit larger basal K^+^ outward and smaller inward currents under 5 kPa O_2_ compared to standard hyperoxic (18 kPa) culture conditions. Outward currents were potentiated by NO only under hyperoxia. Human cerebral microvascular EC (hCMEC/D3) showed larger outward currents under 5 kPa O_2_ which were further potentiated by NO. Pharmacological isolation of different K^+^ currents using tetraethylammonium, TRAM-34 and apamin revealed differential effects in EC adapted to 5 kPa or 18 kPa O_2_. Under 5 kPa O_2_, both cell types show greater contributions of TEA-sensitive currents and in addition hCMEC/D3 cells exhibit higher proportions of TRAM-34 and apamin-sensitive currents under 5 kPa O_2_. In HUVEC, changes in half-activation voltage and hyperpolarized membrane potentials were detected only under hyperoxic conditions following NO exposure, with both cell types exhibiting altered current activation kinetics of outward and inward currents. Notably, expression of KCa3.1, KCa1.1, KCa2.3 and Kir6.1 channels was unaffected by O_2_, suggesting that changes in whole-cell currents in both EC types were due to channel modulation. Thus, our findings reveal that physiological O_2_ tension shapes the electrophysiological phenotype of human EC by modulating K^+^ channel function and NO responsiveness. The novel insights into the modulation of EC K^+^ channels by O_2_ has implications for the regulation of vascular tone and design and use of experimental models *in vitro* for high throughput drug discovery and clinical translation.

## Highlights

• Adaptation to 5 kPa O_2_ enhances basal whole-cell currents in HUVEC and hCMEC/D3.
• NO augments currents in HUVEC and hCMEC/D3 only in hyperoxia and physioxia, respectively.
• BK, SK and IK channels contribute differentially to whole-cell currents.
• Critical importance of physioxia *in vitro* studies to inform vascular modeling and drug screening.

## Graphical abstract

**Figure:**
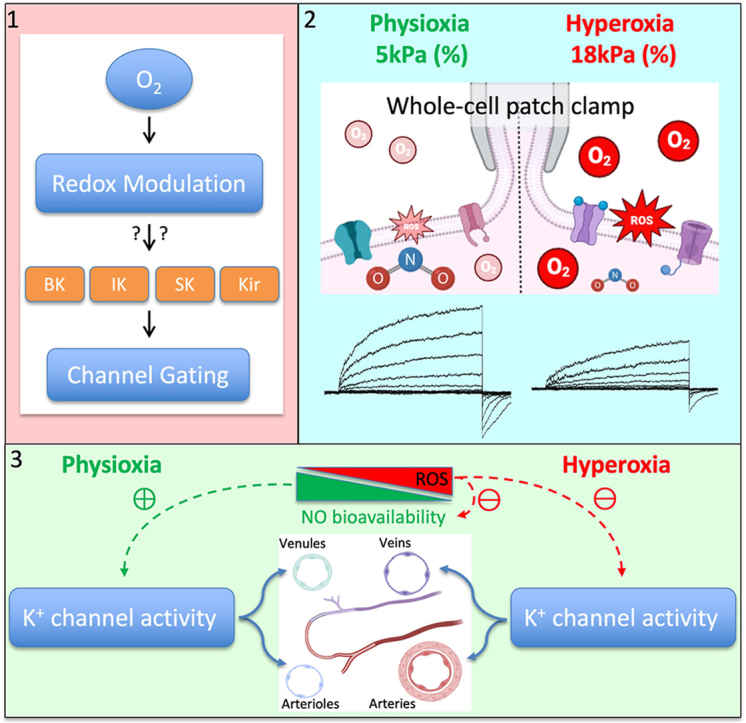

## 1 Introduction

Our current knowledge of the mechanisms underlying endothelial function is derived primarily from cell models maintained under atmospheric O_2_ (∼21 kPa O_2_), known to induce oxidative distress and NRF2 transcriptional activation of antioxidant genes [[1], [2], [3]]. Under physiological conditions, endothelial cells (EC) and most other cell types reside in low-O_2_ environments (3–13 kPa) *in vivo* [1,2]. Recent evidence established that the phenotype and function of cells cultured under physiological O_2_ levels (∼5 kPa, ‘physioxia’) differ significantly from cells maintained under atmospheric O_2_ [1,2,[4], [5], [6], [7], [8], [9], [10], [11]], questioning the validity of studies performed under atmospheric O_2_. Similar to the feto-placental circulation experiencing low *in vivo* O_2_ environments [1,2], the brain microvasculature resides in O_2_ levels of ∼8 kPa in rat and mouse [12,13]. These data indicate that under physiological *in vivo* conditions, macro- and microvascular endothelial cells are exposed to significantly lower O_2_ levels.

The vascular endothelium regulates the contractile state of the vessel wall through the synthesis and release of constrictor and dilatory mediators. Stimulus-induced vasodilation occurs principally *via* mechanisms that involve release of NO or EDRF [14,15] along with non-NO, endothelium-derived hyperpolarising factors (EDHF) [16], requiring controlled regulation of K^+^ channels by Ca^2+^, ATP, redox and NO signalling [[17], [18], [19], [20]]. These mechanisms regulate K^+^ channels in various cell types [[21], [22], [23], [24]] including HUVEC [25], microvascular brain EC and neurons [22,[26], [27], [28]]. HUVEC express various K^+^ channels involved in setting membrane potentials critical for vasoactive responses which are mediated by Ca^2+^-activated BK, IK and SK as well as ATP-sensitive, inward rectifying K^+^ channels (K_IR_/K_ATP_) [[29], [30], [31]], noting that direct and indirect regulation is highly redox sensitive [18,32,33].

Robust NO production is mediated *via* increases in intracellular Ca^2+^ and InsP_3_ signalling in both HUVEC [34,35] and hCMEC/D3 cells [36,37]. hCMEC/D3 cells express PIEZO and TRP4 channels and InsP_3_ receptors that regulate release intracellular Ca^2+^ and show adenosine-mediated regulation of cyclic nucleotide-gated (CNG) channels [36,38], making it a relevant model of human brain microvascular endothelial cells to study nitrergic signalling and K^+^ channel function.

As endothelial cells under physiological O_2_ tension exhibit lower redox distress and enhanced NO bioavailability [5,6,11,39], this study investigates for the first time basal and NO-modulated whole-cell K^+^ currents in human EC adapted for 5 days to hyperoxia (18 kPa) or physiological normoxia (5 kPa). We report increased basal outward currents under physioxia in both cell types and a differential response to NO exposure on outward currents between both cell types. HUVEC only show a current increase under hyperoxic conditions, whereas hCMEC/D3 show a current potentiation by NO only under physioxia, highlighting differential regulation in NO and/or redox signalling cascades [5,40]. In the presence of K^+^ channel inhibitors tetraethylammonium (TEA), TRAM-34 or apamin under 5 kPa O_2_, both cell types exhibited greater contribution of TEA-sensitive currents, and notably hCMEC/D3 show larger TRAM-34 and apamin sensitive currents only at 5 kPa O_2_. Inward currents were only detected in HUVEC in which NO enhanced current amplitudes under physioxia. Thus, our findings have significant implications for selecting live cell models *in vitro* for drug discovery and clinical translation.

## 2 Materials and methods

### 2.1 Cell culture

Human umbilical vein endothelial cells (HUVEC, PromoCell, Germany) were cultured in endothelial cell medium (ECM) supplemented with 5 % fetal bovine serum (FBS), 1 % endothelial cell growth supplement (ECGS) and 1 % penicillin (100U/ml)/streptomycin (100 μg/ml) (ScienCell Research Laboratories, USA). Human brain microvascular endothelial cells (hCMEC/D3 cells, Cedarlane Laboratories, Canada) were cultured in 1 % rat-tail collagen I coated substrates (Sigma) in EBM phenol-red free basal cell media (Lonza, Switzerland) supplemented with EGM-2MV growth factors, 0.025 % (v/v) rhEGF, 0.025 % (v/v) VEGF, 0.025 % (v/v) IGF, 0.1 % (v/v) rhFGF, 0.1 % (v/v) ascorbic acid, 0.04 % (v/v) hydrocortisone, 2.5 % FBS (Lonza, Switzerland) and 1 % penicillin (100U/ml)/streptomycin (100 μg/ml).

HUVEC and hCMEC/D3 cell monolayers were maintained for at least 5 days in an O_2_-controlled Scitive dual or InVivO_2_®400 workstation (Baker, USA), gassed to 18 kPa or 5 kPa O_2_ under 5 % CO_2_ at 37^o^C. We previously established that lowering ambient O_2_ in the workstation from 18 kPa to 5 kPa rapidly upregulates HIF1-α mRNA expression with protein expression of HIF1-α and its targets only returning to baseline after 5 days adaptation [11]. HUVEC and hCMEC/D3 cells monolayers were passaged with trypsin, and importantly all cell culture protocols and experiments were conducted within the O_2_-controlled workstations to avoid re-exposure of cells to atmospheric oxygen and stabilization of HIF-1α upon lowering ambient O_2_ levels from 18 kPa to 5 kPa [4,11].

### 2.2 Whole-cell patch clamp recordings under defined oxygen levels

A Nanion Port-a-Patch head stage housed within an O_2_ controlled workstations was used for whole-cell recordings, using NPC-1 chips at 3.5–5.0MΩ with series resistances of ∼10–15MΩ under defined O_2_ levels. An EPC 10 patch clamp amplifier including PatchMaster v2x73 (HEKA) was used for data acquisition and analysis (voltage clamp and current clamp), cell suspensions were prepared as previously reported and all whole-cell currents were recorded using the P/L leak subtraction protocol [41]. During all recordings, step pulses were applied in 10/20 mV increments from −120 mV to +50/+60 mV with the holding potential of −60 mV. Conductance values were obtained from G = I/(V − Vrev), where I is the peak current during the test depolarisation (V), and Vrev is the potassium reversal potential. Data were normalised to maximum peak conductance (Gmax) and fit to the following form of a two-state Boltzmann distribution: G/Gmax = (1 + exp (V_1/2_ − V)/k), where V_1/2_ is the half-activation potential, V is the test potential, and k is the slope factor. Activation kinetics were measured by fitting the activation current trace at +50 mV from the beginning of the depolarizing step until the peak, using a double exponential function to obtain τ_2_ values.

Experiments were performed in HEPES buffered standard recording solution at ∼37^o^C under a 5 kPa or 18 kPa O_2_ environment (Patch clamp: Extracellular recording solution: 2 mM CaCl_2_, 5 mM KCl, 138 mM NaCl, 1 mM MgCl_2_, 10 mM HEPES, 10 mM d-Glucose and pH adjusted to 7.4 using 1 M NaOH; intracellular solution: 10 mM EGTA, 10 mM HEPES, 10 mM KCl, 10 mM NaCl, 110 mM kF, pH 7.2 adjusted with KOH. Experiments where performed under minimal intracellular Ca^2+^ levels (∼15–20 nM) [42] at which the movements of the voltage sensing domain within BK channels, one of the largest K^+^ conductances in HUVEC, generates curents between 0 and +300 mV [43,44]. Cells were treated with NO donors (400 μM SNP (Sigma) or 400 μM NOC-7 (Santa Cruz Biotechnology)) and K^+^ currents recorded within 10min. Solutions for electrophysiological recordings were from Nanion Technologies. TEA (10 mM) was from Scientific Laboratory Supplies Ltd, UK. TRAM-34 (5 μM) was from Cambridge Bioscience Ltd, UK. Apamin (100 nM) was from Smartox Biotechnology, France.

### 2.3 Immunoblotting

Whole cell lysates were collected from HUVEC or hCMEC/D3 cells maintained within the workstations for 5 days under 18 kPa or 5 kPa O_2_, using SDS lysis buffer supplemented with protease inhibitor cocktail (Sigma-Aldrich, UK). Lysates were subjected to gel electrophoresis, electro-transferred onto polyvinylidene difluoride membranes (Millipore, Sigma, USA), probed with primary and HRP-conjugated secondary antibodies (1: 2000, Millipore, Sigma, USA): Anti-Kir6.1/KCNJ8 (1:1000, Abcam, UK), Anti-KCNN3 (KCa2.3, SK3) (C-term) (1:200, Alomone Labs, Israel) and β-actin (1:8000, Sigma-Aldrich, UK) and analyzed by enhanced chemiluminescence (Millipore, Sigma, USA). All images were captured using a G:Box system (Syngene, UK) and densitometric analysis conducted using ImageJ software (National Institute of Health, USA).

### 2.4 Immunocytochemistry

To assess expression of KCNN4 (KCa3.1, SK4) and KCNMA1 (KCa1.1, BK_Ca_), HUVEC or hCMEC/D3 were pre-adapted for 5 days to 18 kPa or 5 kPa O_2_ and then seeded into black clear bottom 96-well plates for 48h. Immunofluorescence analysis was performed using a primary Anti-KCNN4 (KCa3.1, SK4) (1:20, Alomone Labs, Israel), Anti-KCNMA1 (KCa1.1, BKCa) (1:200, Alomone Labs, Israel) and goat anti-Mouse Alexa Fluor™ 488 or goat anti-Rabbit Alexa Fluor™ 555 conjugated secondary antibody (1:1000, Invitrogen, USA). Cell nuclei were stained with DAPI (1:10000, Sigma, UK). Fluorescence was visualized at × 40 magnification using a fluorescence microscope (EVOS M7000, USA), with images quantified using plate reader (CLARIOstar, BMG Labtech, Germany) as the ratio of protein targeted fluorescence intensity over DAPI fluorescence.

### 2.5 Statistical analysis

Data denote the mean ± S.E.M. of 3–6 independent HUVEC and hCMEC/D3 cell cultures and were analyzed using Graphpad Prism 10.4. Significance was assessed using either an unpaired Student's *t*-test or one- or two-way ANOVA followed by a Bonferroni post-hoc test where appropriate, with *P* < 0.05 considered significant. Where possible, all data underwent normality tests showing normal distributions (Shapiro–Wilk test) and homogeneity of variance (Bartlett's test) which was similar between groups that were statistically compared. Replicates denote biological replicates.

## 3 Results

### 3.1 Whole-cell voltage clamp recordings in HUVEC adapted to 5 kPa or 18 kPa O_2_

**Fig. 1:**
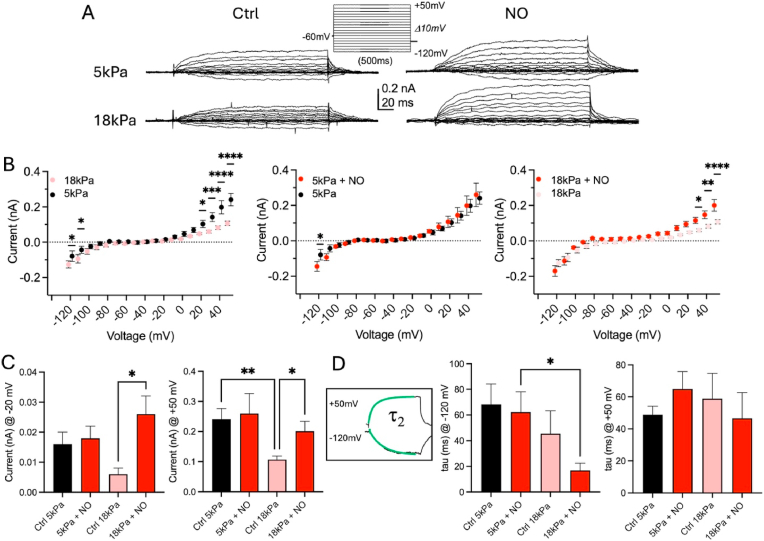
Whole-cell currents in HUVEC adapted to 5 kPa or 18 kPa O_2_. (**A**) Raw current traces under indicated conditions with voltage protocol using the Nanion P-a-P. HUVEC exhibit inward- and outward rectifying K^+^ currents showing differential responses under defined physiological O_2_ levels and following incubation with an NO donor. (**B**) Steady-state current-voltage (IV) relationships differ in HUVEC under indicated conditions (left: F_Treatment_ (17, 738) = 38.02, *P* < 0.0001, F_Voltage_ (1, 738) = 50.83, *P* < 0.0001; middle: F_Treatment_ (17, 558) = 29.60, *P* < 0.0001, F_Voltage_ (1, 558) = 0.3886, *P* = 0.5333; right: F_Treatment_ (17, 648) = 58.70, *P* < 0.0001, F_Voltage_ (1, 648) = 32.51, *P* < 0.0001, two-way ANOVA). Notably, inward currents are smaller under 5 kPa compared to 18 kPa O_2_, whereas outward currents are larger. NO induced stronger relative potentiation of inward and outward currents under 18 kPa O_2_. (**C**) Direct comparisons of current amplitudes at −20 mV and +50 mV under indicated conditions (−20 mV, *P* = 0.0127, +50 mV, *P* = 0.012, one-way ANOVA). (**D**) Exponential fits to the rising phase of current activation. Schematic illustrates the best fit (green line) by a second order exponential with time constants of activation shown in graphs for currents at −120 mV and + 50 mV (*P* = 0.0465, one-way ANOVA). Data denote mean ± S.E.M., n = 7–21 cells per condition.

Whole-cell voltage clamp recordings were obtained in HUVEC and hCMEC/D3 adapted for 5 days to either hyperoxia (18 kPa O_2_) or physiological normoxia (5 kPa O_2_ ‘physioxia’) to determine the regulation of whole-cell currents under O_2_ levels encountered *in vivo*. Under unstimulated conditions at both O_2_ tensions, HUVEC exhibited heterogeneous whole-cell currents with cells displaying either inwardly or outwardly rectifying conductance or showing both inward and outward components of whole-cell currents (holding potential: −60 mV, Fig. 1A), ranging from around −200 pA – 300 pA. These values for whole-cell currents have been previously reported in control HUVEC under comparable recording conditions [[45], [46], [47], [48]] and are described as being generated by K^+^ channels. Following adaptation to 5 kPa O_2_ for 5 days, naïve HUVEC exhibited significantly greater outward currents at voltages above +20 mV compared to 18 kPa culture conditions (+20 mV: *P* = 0.0132, +30 mV: *P* = 0.0002, +40 mV and +50 mV: *P* < 0.0001, two-way ANOVA, Fig. 1A and B) with a threshold potential of activation for outward currents between −40 mV and −10 mV. As capacitance values between both culture conditions did not differ (HUVEC: 5 kPa O_2_: 19.8 ± 2.1 pF *versus* 18 kPa O_2_: 20.6 ± 1.4 pF, *P* > 0.05; hCMEC/D3: 5 kPa O_2_: 13.7 ± 2.3 pF *versus* 18 kPa O_2_: 14.7 ± 3.1 pF, *P* > 0.05 Student's *t*-test), all current values are expressed as absolute non-normalised whole-cell current amplitudes [45,47].

Under physioxia, several signaling pathways display significant differences compared to hyperoxia. Notably, in cells adapted to physioxia reactive oxygen species generation is lower and NO bioavailability are enhanced and agonist (histamine)-stimulated Ca^2+^ entry is reduced [1,2]. These differences led us to explore for the first time whether NO modulates whole-cell currents differentially under 5 kPa or 18 kPa O_2_. In these experiments, currents were recorded under low internal Ca^2+^ concentrations to avoid Ca^2+^-dependent channel modulation and minimise endogenous NO interactions.

### 3.2 NO modulation of whole-cell currents in HUVEC adapted to 5 kPa or 18 kPa O_2_

NO-mediated whole-cell current potentiation was detectable in HUVEC following 18 kPa O_2_ adaptation, with current augmentation at voltages above +30 mV (30 mV: *P* = 0.0483, 40 mV: *P* = 0.0081, 50 mV: *P* < 0.0001, two-way ANOVA, Fig. 1A and B). However, under 5 kPa O_2_, we did not detect any NO effects on outward currents, suggesting that under this condition whole-cell outward currents are already maximally enhanced to physiological levels and thus were not further increased in response to NO (*P* > 0.05, two-way ANOVA, Fig. 1A and B). In contrast, inward currents were larger at negative potentials under 18 kPa compared to 5 kPa O_2_ (−110 mV: *P* = 0.0216, −120 mV: *P* = 0.0342, two-way ANOVA, Fig. 1A and B).

Our findings indicate that whole-cell K^+^ current regulation in EC *in vitro* differs between physiological and hyperoxic O_2_ levels, and that differential responses to NO application in EC under 5 kPa and 18 kPa O_2_ markedly affect inward and outward currents *via* different pathways. A direct comparison of whole-cell current amplitudes across all four conditions at a physiologically relevant voltage close to resting membrane potential of ∼ −20 mV and at maximal depolarisation at 50 mV, revealed significant differences between basal and NO stimulation only under hyperoxic 18 kPa O_2_ (Fig. 1C, −20mV: *P* = 0.0012; 50 mV: *P* = 0.0494, one-way ANOVA). Under 5 kPa O_2_, outward whole-cell currents at 50 mV are already increased in unstimulated EC compared to 18 kPa O_2_ (Fig. 1C, *P* = 0.0035, one-way ANOVA) indicating a possible endogenous NO-mediated channel activation due to reduced redox distress and increased NO bioavailability [6,40].

Many K^+^ channels, including voltage-gated (Kv) channels, BK channels and GIRK channels can be modified by oxidizing agents *in vivo* and *in vitro* [[49], [50], [51], [52], [53], [54]]. This redox modulation affects open probabilities (and whole-cell current amplitudes) but also time constants of channel activation. Hyperoxic (∼18 kPa) exposure of cells elevates levels of redox active molecules and affects various signalling cascades [1,2,55]. To identify potential changes in whole-cell current activation kinetics between different O_2_ adaptation regimes, we measured the time constants of activation of step depolarisation-induced currents by fitting the rising phases of the currents evoked by a 500 ms voltage step to −120 mV and +50 mV (holding potential: −60 mV). The currents were best fitted with a second-order exponential function. Our data revealed differences in activation kinetics of inward whole-cell (K^+^) currents between 5 kPa and 18 kPa following NO exposure, which may suggest differences in their redox-mediated regulation (Fig. 1D).

**Fig. 2:**
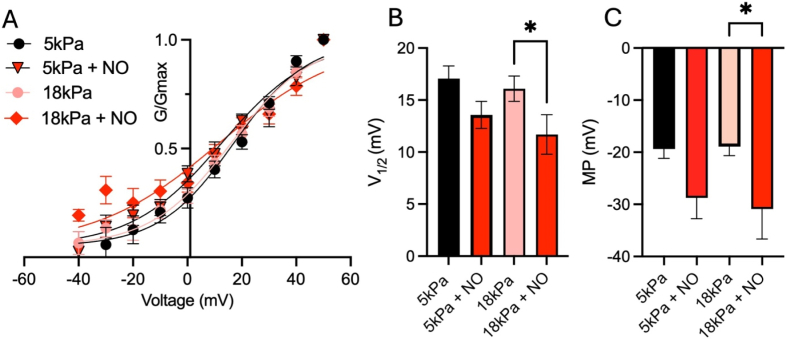
NO application modulates channel activation and membrane potentials differently in HUVEC adapted to 5 kPa or 18 kPa O_2_. (**A, B**) Boltzmann fits to G/G_max_ for indicated conditions. Data following NO treatments suggests a leftward shift of half activation voltages (V_1/2_) at 18 kPa pericellular O_2_ (5 kPa: 17.0 ± 1.2 mV, 5 kPa + NO: 13.5 ± 1.3 mV; 18 kPa: 16.1 ± 1.2 mV, 18 kPa + NO: 11.7 ± 1.9 mV, n = 9–23, one-way ANOVA, *P* = 0.0061, n = 7–21 cells per condition). (**C**) Membrane potentials were recorded in control HUVEC under indicated conditions. NO application induces a hyperpolarization in cells adapted to hyperoxic 18 kPa O_2_ (one-way ANOVA, *P* = 0.0176, n = 5–8 cells per condition). Data denote mean ± S.E.M.

Redox modulation can shift the conductance-voltage (GV) relationship of BK channels [56]. To test whether under our conditions we observe such changes, we calculated half-activation voltages (V_1/2_) using a Boltzmann fit to whole-cell currents and plotted G/G_max_ in control HUVEC under both oxygen levels and following incubation with NO (Fig. 2A and B). The estimated V_1/2_ (18 kPa: 16.1 ± 1.2 mV, 5 kPa:17.1 ± 1.2 mV, 18 kPa + NO: 11.7 ± 1.9 mV, 5 kPa + NO: 13.5 ± 1.3 mV, *P* = 0.0316, one-way ANOVA) and slope k (18 kPa: 14.8 ± 1.1, 5 kPa:13.7 ± 1.1, 18 kPa + NO: 22.7 ± 2.1, 5 kPa + NO: 16.5 ± 1.2) values lie within the range of reported values for BK channels at low physiological intracellular Ca^2+^ levels (∼15–20 nM) [42,57,58], with our values most likely reflecting the combined whole-cell current activation consisting of different K^+^ channels. We found that under hyperoxic O_2_ conditions, NO application caused a leftward shift of V_1/2_ mirroring the augmented currents detected under these conditions (Fig. 2B, *P* = 0.0316, one-way ANOVA).

K^+^ channels are responsible for setting EC membrane potentials which ultimately determine their physiological function. As NO regulates K^+^ channels, we next tested whether differential NO regulation of whole-cell currents under both O_2_ levels affects membrane potentials differently. The measured resting membrane potentials in unstimulated HUVEC under both O_2_ levels were unchanged (Fig. 2C, Control: 5 kPa: −19.4 ± 1.8 mV and 18 kPa: −18.9 ± 1.7 mV, *P* = 0.1555, one-way ANOVA) and comparable with previously reported values in HUVEC [48] and other endothelial cells [59]. Membrane potentials were only hyperpolarized in HUVEC adapted to 18 kPa O_2_ following NO application (Fig. 2C, NO: 5 kPa: −28.8 ± 4.0 mV and 18 kPa: −30.9 ± 5.7 mV, *P* = 0.0247, one-way ANOVA), consistent with K^+^ channel activation only under these conditions (Fig. 1A and B). Although NO induces hyperpolarization under both O_2_ conditions (Fig. 2C), the underlying mechanisms differ. Under 18 kPa O_2_, hyperpolarization reflects a robust increase in outward K^+^ currents (Fig. 1), whereas under 5 kPa, currents were not further potentiated by NO, suggesting that channels are already maximally activated under physioxia. The small V_1/2_ shift under 5 kPa (Fig. 2B) likely represents subtle gating changes without functional impact on whole-cell currents.

### 3.3 Effects of oxygen and NO on whole-cell currents in hCMEC/D3 cells

hCMEC/D3 cells exhibit Ca^2+^-dependent production of vasoactive signaling molecules such as NO [37] which regulate microvascular and blood-brain-barrier function. Increases in intracellular Ca^2+^ stimulate eNOS in human cerebrovascular EC. To establish whether O_2_-dependent regulation of whole-cell channel properties observed in HUVEC are similar in hCMEC/D3 cells, we measured basal channel activities under both O_2_ levels and examined whether responses to NO application differ between hyperoxia and physioxia.

**Fig. 3:**
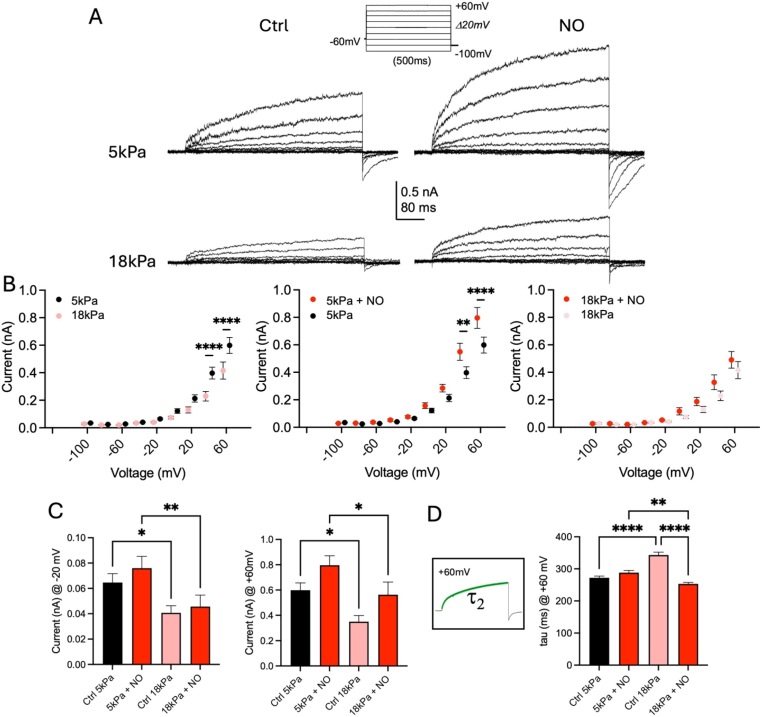
Whole-cell currents in human brain microvascular endothelial cells adapted to 5 kPa or 18 kPa O_2_. (**A**) Raw current traces under indicated conditions with voltage protocol using the Nanion P-a-P. hCMEC/D3 cells exhibit outward rectifying K^+^ currents showing differential responses under defined O_2_ levels and following incubation with an NO donor. (**B**) Steady-state current-voltage (IV) relationships differ between hCMEC/D3 cells recorded under indicated conditions (left: F_Treatment_ (8, 243) = 84.08, *P* < 0.0001, F_Voltage_ (1, 243) = 23.33, *P* < 0.0001; middle: F_Treatment_ (8, 252) = 119.3, *P* < 0.0001, F_Voltage_ (1, 252) = 13.89, *P* = 0.0002; right: F_Treatment_ (9, 268) = 58.59, *P* < 0.0001, F_Voltage_ (1, 268) = 5.876, *P* = 0.0160, two-way ANOVA). Notably, outward currents are larger under 5 kPa compared to 18 kPa O_2_. NO induces a stronger potentiation of outward currents under 5 kPa O_2_. **C**, Direct comparisons of current amplitudes at −20mV and +60 mV under indicated conditions (−20 mV: ∗*P* = 0.0426, ∗∗*P* = 0.0087; +60 mV: 5 kPa *versus* 18 kPa: *P* = 0.0318, 5 kPa + NO *versus* 18 kPa + NO: *P* = 0.0309, one-way ANOVA). **D**, Exponential fits to the rising phase of current activation. Schematic illustrates the best fit (green line) by a second order exponential with time constants of activation shown in graphs for currents at +60 mV (∗∗*P* = 0.0035, ∗∗∗∗*P* < 0.0001, one-way ANOVA, n = 14–15 cells per condition). Data denote mean ± S.E.M.

As shown in Fig. 3A, hCMEC/D3 exhibited only outwardly rectifying currents, confirming findings in a previous report under 18 kPa O_2_ [41]. Naïve hCMEC/D3 displayed larger outward currents under 5 kPa O_2_ at voltages above +40 mV (40 mV: *P* < 0.0001, 60 mV: *P* < 0.0001, two-way ANOVA). Application of NO increased outward currents only under 5 kPa (40 mV: *P* = 0.0053 and 60 mV: *P* < 0.0001, two-way ANOVA). However, under 18 kPa O_2_, NO did not induce a current potentiation at any holding voltage (two-way ANOVA, Fig. 3A and B). A one-way ANOVA comparison of current amplitudes at −20 mV and 60 mV between all four conditions revealed significant increases in basal and NO-stimulated currents in cells adapted to 5 kPa O_2_ (Fig. 3C), indicating an augmented potentiation of K^+^ currents under both conditions. Whether direct or indirect signaling *via* redox regulation [[1], [2], [3]] underlies this observation remains to be elucidated.

Further evidence for differences in macro- and microvascular EC whole-cell currents was found by assessing current activation time constants. We applied fits with a second-order exponential function to outward whole-cell currents at +60 mV and confirmed changes in current activation kinetics at rest between O_2_ levels and following NO application under 5 kPa and 18 kPa O_2_ (NO: 5 kPa *versus* 18 kPa: *P* = 0.0035, one-way ANOVA, Fig. 3D).

### 3.4 K^+^ channel characterization in HUVEC and hCMEC/D3 adapted to 5 kPa or 18 kPa O_2_

**Fig. 4:**
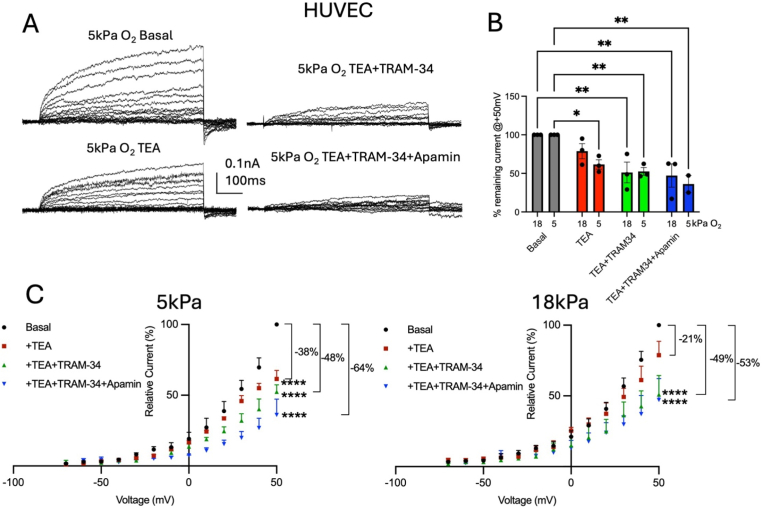
K^+^ channel contributions to whole-cell current vary between cells adapted to 5 kPa or 18 kPa O_2_. (**A)** Raw current traces under indicated conditions recorded from HUVEC exhibit outward rectifying whole-cell currents. (**B)** Comparisons of normalised current amplitudes (relative to current at +50 mV) under indicated conditions show current inhibition relative to basal control from cells cultured at 5 kPa or 18 kPa using 10 mM TEA, 5 μM TRAM-34, 100 nM apamin (5 kPa: Basal vs. TEA: *P* = 0.0381, Basal vs. TEA + TRAM-34: *P* = 0.0095, Basal vs. TEA + TRAM-34+apamin: *P* = 0.0022; 18 kPa: Basal vs. TEA: *P* = 0.03723, Basal vs. TEA + TRAM-34: *P* = 0.0076, Basal vs. TEA + TRAM-34+apamin: *P* = 0.0042; two-way ANOVA, data denote mean ± S.E.M.). (**C**) Steady-state current-voltage (IV) relationships showing differential effects of TEA, TRAM-34 and apamin in HUVEC adapted to either O_2_ level (5 kPa O_2_: F_Treatment_ (3, 90) = 49.88, *P* < 0.0001, F_Voltage_ (12, 90) = 131.0, *P* < 0.0001; 18 kPa O_2_: F_Treatment_ (3, 104) = 15.50, *P* < 0.0001, F_Voltage_ (12, 104) = 55.47, *P* < 0.0001, n = 2–3 cells each, two-way ANOVA, data denote mean (+S.E.M.).

As HUVEC express BK, IK and SK channels as well as inward rectifier K^+^ channels [30,46,48], we examined the effects of sequentially blocking BK, IK and SK channels using TEA (10 mM), TRAM-34 (5 μM) and apamin (100 nM), respectively, in EC adapted to 5 kPa or 18 kPa O_2_. Our recordings show a functional contribution of BK, IK and SK channels to the overall whole-cell current in both cell types (Fig. 4). Although HUVEC also express TRPV1 [60], PIEZO1 [61] and chloride channels [62,63] which are sensitive to flow, changes in protons/pH and are activated by volume changes and Ca^2+^, we have not assessed whether they are functionally involved under our recording conditions. hCMEC/D3 express mechano-sensitive Piezo1 and TRPV4 and cyclic nucleotide gated (CNG) channels [36,38], however, an electrophysiological characterisation of whole-cell currents in hCMEC/D3 has not been established. Our data show that whole-cell outward currents were sensitive to all three inhibitors under both O_2_ culture regimes (Fig. 4) however, the relative proportions of blocked currents differed between hyperoxic and physioxia conditions.

In HUVEC, TEA (10 mM) application shows a significant reduction in whole-cell currents at 50 mV only under 5 kPa O_2_ (data expressed as normalised current relative to maximal amplitude at 50 mV, 5 kPa and 18 kPa: TEA: 62 ± 5 % *P* = 0.0381 and 79 ± 8 % *P* = 0.3723, TEA + TRAM-34: 52 ± 4 % *P* = 0.0095 and 51 ± 11 % *P* = 0.0076, TEA + TRAM-34+apamin: 36 ± 6 % *P* = 0.0022 and 47 ± 12, *P* = 0.0042, n = 2–3 each, two-way ANOVA, Fig. 4A and B). The complete IV curves in Fig. 4C indicate the relative reductions in currents across all voltages (all currents were normalised to the maximal amplitude of basal controls at 50 mV, comparisons of inhibitions at 5 kPa and 18 kPa: TEA: 38 ± 5 % *P* < 0.0001 and 21 ± 4 % *P* = 0.0507, TEA + TRAM-34: 48 ± 4 % *P* < 0.0001 and 49 ± 10 % *P* < 0.0001, TEA + TRAM-34+apamin: 64 ± 6 % *P* < 0.0001 and 53 ± 12, *P* < 0.0001, each n = 2–3 each, two-way ANOVA).

**Fig. 5:**
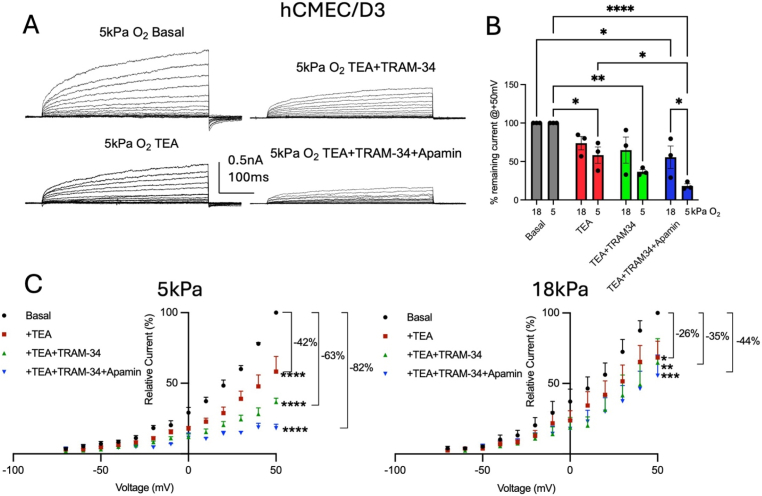
K^+^ channel contributions to whole-cell current vary between cells adapted to 5 kPa or 18 kPa O_2_. (**A)** Raw current traces under indicated conditions recorded from hCMEC/D3 cells exhibit outward rectifying whole-cell currents. (**B**) Comparisons of normalised current amplitudes (relative to current at +50 mV) under indicated conditions show current inhibition relative to basal control from cells cultured at 5 kPa or 18 kPa using 10 mM TEA, 5 μM TRAM-34, 100 nM apamin (5 kPa: Basal vs. TEA: *P* = 0.0375, Basal vs. TEA + TRAM-34: *P* = 0.0013, Basal vs. TEA + TRAM-34+apamin: *P* < 0.0001; 18 kPa: Basal vs. +TEA + TRAM-34+apamin: *P* = 0.0244, two-way ANOVA, data denote mean ± S.E.M.). (**C**) Steady-state current-voltage (IV) relationships showing differential effects of TEA, TRAM-34 and apamin in hCMEC/D3 cells adapted to either O_2_ level (5 kPa O_2_: F_Treatment_ (3, 102) = 173.0, *P* < 0.0001, F_Voltage_ (12, 102) = 131.4, *P* < 0.0001; 18 kPa O_2_: F_Treatment_ (3, 103) = 14.96, *P* < 0.0001, F_Voltage_ (12, 103) = 38.59, *P* < 0.0001, n = 3 cells each, two-way ANOVA, data denote mean (+S.E.M.).

When assessing relative effects of the three K^+^ channel inhibitors on the overall current under both O_2_ adaptation regimes in hCMEC/D3 cells, we noted that under 5 kPa O_2_ cells also exhibited a larger proportion of currents sensitive to all blockers (82 %) compared to 44 % in hyperoxic cultures (Fig. 5). At 5 kPa O_2_, each inhibitor (10 mM TEA, 5 μM TRAM-34, 100 nM apamin) caused a significant reduction in whole-cell current amplitudes at 50 mV relative to basal control currents (data expressed as normalised current relative to maximal amplitude at 50 mV, 5 kPa and 18 kPa: TEA: 58 ± 9 % *P* = 0.0291 and 74 ± 7 % *P* = 0.2394, TEA + TRAM-34: 37 ± 2 % *P* = 0.0011 and 65 ± 14 % *P* = 0.0743, TEA + TRAM-34+apamin: 18 ± 2 % *P* < 0.0001 and 56 ± 12, *P* = 0.0193, n = 3 each, two-way ANOVA, Fig. 5B). Fig. 5C shows normalised IV curves illustrating the sequential block of different K^+^ channels (5 kPa O_2_: current inhibition measured at 50 mV: TEA: 42 ± 8 % *P* = 0.0375, TEA + TRAM-34: 63 ± 2 % *P* = 0.0013, TEA + TRAM-34+apamin: 80 ± 2 %, *P* < 0.0001, n = 3 each, two-way ANOVA). However, in cells adapted to 18 kPa, TEA, TEA + TRAM-34 and TEA + TRAM-34+apamin also reduced whole-cell currents amplitudes but only between 30 mV and 50 mV and to a smaller degree (Fig. 5B, measured at 50 mV: TEA: 26 ± 7 %, *P* = 0.03, TEA + TRAM-34: 35 ± 13 %, *P* = 0.0038, TEA + TRAM-34+apamin: 44 ± 11 % *P* = 0.0001, Fig. 5B, n = 3 each, two-way ANOVA).

Thus, our findings in HUVEC and hCMEC/D3 suggest that functional contribution, composition and/or expression of K^+^ channels differs in cells adapted to physiological or hyperoxic O_2_ levels, leading to changes in the observed electrophysiological profiles. To investigate whether the expression of different K^+^ channels was affected by pericellular oxygen, we assessed protein expression by immunoblotting and immunocytochemistry in both cell types.

### 3.5 Unaltered K^+^ channel protein expression following adaptation of endothelial cells to physioxia

**Fig. 6:**
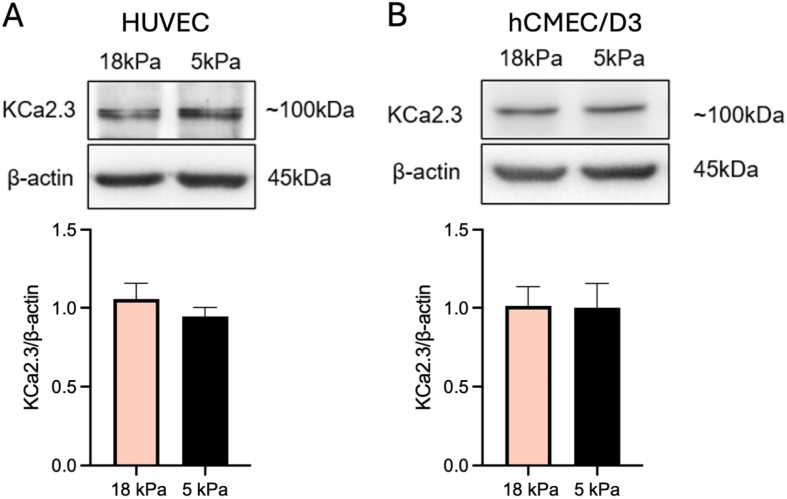
Expression of KCa2.3 in human umbilical vein and human brain microvascular endothelial cells adapted to 5 kPa *versus* 18 kPa O_2_. (**A, B**) Immunoblot analysis of KCa2.3 (SK3) channel expression in human umbilical vein (HUVEC) and brain microvascular (hCMEC/D3) endothelial cells adapted for 5 days to 5 kPa or 18 kPa O_2_ reveals no difference (n = 3 independent cultures, Student's *t*-test, *P* > 0.05). Data denote mean ± S.E.M.

HUVEC or hCMEC/D3 cells cultured under standard hyperoxia exhibited altered whole-cell K^+^ currents which could be the consequence of changes in K^+^ channel activities directly or indirectly *via* redox modulation [28,64,65] or K^+^ channel protein expression. Direct modulation of various K^+^ channels by NO has been widely reported [49,[66], [67], [68], [69], [70]]. To confirm potential changes in the expression of K^+^ channels in both cell types, we assessed protein expression of large Ca^2+^-activated BK, IK and SK channels, identifiedin HUVEC and hCMEC/D3 [60,71,72]. No differences in expression of SK3 were detected between physiological and hyperoxic O_2_ levels in either cell type (Fig. 6, ratio channel protein/β-actin, HUVEC: SK3: 5 kPa: 0.95 ± 0.06, 18 kPa: 1.06 ± 0.09, *P* = 0.37; hCMEC/D3: SK3: 5 kPa: 1.01 ± 0.15, 18 kPa: 1.0 ± 0.12, *P* = 0.96; Student's *t*-test), suggesting that our recorded changes in ion channel activity cannot be explained by changes in SK channel expression.

**Fig. 7:**
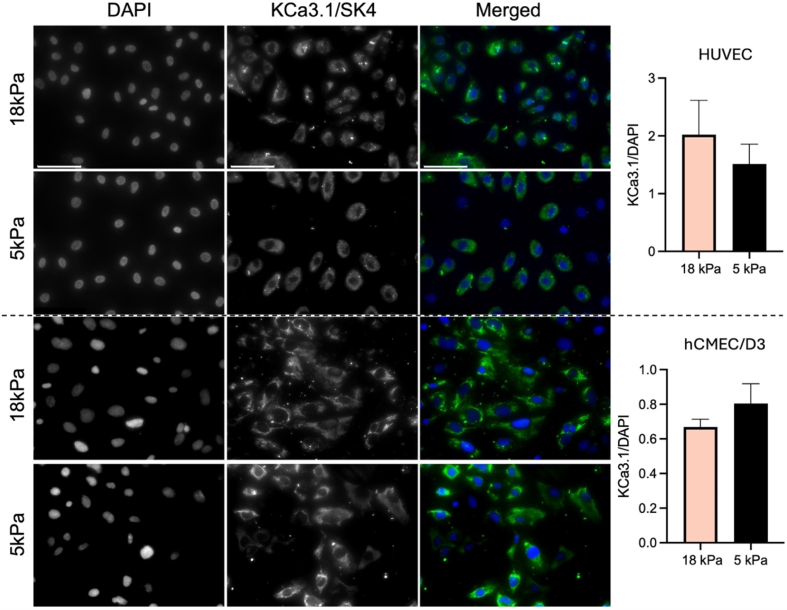
Expression of KCa3.1 channels in human umbilical vein and human brain microvascular endothelial cells adapted to 5 kPa *versus* 18 kPa O_2_. Representative immunocytochemical images of HUVEC (top) and hCMEC/D3 (bottom) immunostained for KCa3.1 (SK4) channel protein with quantifications of fluorescence signals normalised to DAPI in HUVEC and hCMEC/D3. Data denote mean ± S.E.M., n = 6 independent cultures, Student's *t*-test, *P* > 0.05).

**Fig. 8:**
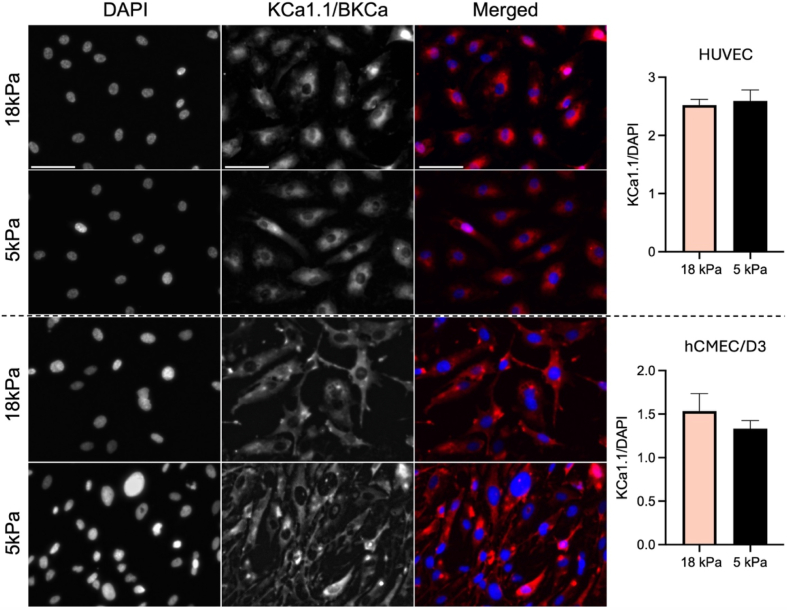
Expression of KCa1.1 channels in human umbilical vein and human brain microvascular endothelial cells adapted to 5 kPa *versus* 18 kPa O_2_. Representative immunocytochemical images of HUVEC (top) and hCMEC/D3 (bottom) immunostained for KCa1.1 (BK) channel protein with quantifications of fluorescence signals normalised to DAPI in HUVEC and hCMEC/D3. Data denote mean ± S.E.M., n = 4 independent cultures, Student's *t*-test, *P* > 0.05).

Additional immunocytochemical analyses were performed to investigate expression of KCa3.1/SK4 and KCa1.1/BK. We measured KCa3.1 and KCa1.1 fluorescence signals in HUVEC and hCMEC/D3 cultures adapted to 5 kPa or 18 kPa O_2_ and found no differences in either channel expression in both cell types under hyperoxic or physioxia culture conditions (Fig. 7, Fig. 8; *P* > 0.05, Student's *t*-test), suggesting again that the observed differences in electrophysiological data are likely due to a K^+^ channel regulation rather than expression changes. Although functional differences in inward currents in HUVEC were small, we decided to also test for protein expression of KCNJ8, a subunit forming the functional Kir6.1 channel. Expression of this subunit in HUVEC was also not affected by culture conditions (Suppl. Fig. 1).

## 4 Discussion

The sensitivity of K^+^ channels to redox signaling has important implications for endothelial physiology. Elevated O_2_ levels are associated with increased generation of reactive oxygen species [2] and reduced NO bioavailability [6,11], which may compromise NO-mediated regulation of channel activities and ultimately EC function [73]. To our knowledge, our study is the first to investigate the effects of long-term adaptation (5 days) of macro- and microvascular EC to physioxia (5 kPa) on basal and NO-modulated K^+^ channel activities. Endothelial cells were adapted to 5 kPa O_2_ to ensure a physiological O_2_ and redox phenotype in the absence of HIF-1α stabilization. We previously established that lowering pericellular O_2_ levels induces rapid and relatively long-lasting stabilization of HIF-1α and its targets which return to baseline levels within 5 days [4,11]. Nevertheless, a time-course analysis of the effects of lowering pericellular O_2_ tension on ion channel activity would be of interest in future studies. We identified discrete changes under physioxia, noting the both cell types responded with larger outward whole-cell currents. HUVEC showed an additional current potentiation following NO exposure only under 18 kPa O_2_, whereas in hCMEC/D3 cells NO-mediated current potentiation was detectable only under 5 kPa O_2_. Although both cell types generate NO, this difference may reflect distinct cellular signaling defined by their *in vivo* origin, e.g. fetal macrovascular *versus* brain microvascular origin. Functionally, hCMEC/D3 are involved in neurovascular coupling, translating neuronal activity into increased cerebral blood flow and blood-brain barrier function and are thus targets of NO. HUVEC generate NO to modulate vascular tone in response to shear stress and vasoactive mediators and are predominantly a source of NO production.

While our findings demonstrate oxygen-dependent modulation of K^+^ channel activity in both cell types, differences in media composition may also contribute to functional variability. Future studies should examine whether medium complexity influences endothelial ion channel regulation under physiological O_2_. Although we did not directly measure eNOS expression or activity in this study, our previous findings established that adaptation of endothelial cells to physiological O_2_ does not alter eNOS expression/activity but enhances intracellular NO bioavailability due to significantly lower intracellular O_2_ levels [6,11]. Thus, increased NO bioavailability most likely contributes to the differential NO responsiveness observed in HUVEC and hCMEC/D3 under 5 kPa *versus* 18 kPa O_2_.

The majority of cell culture studies have been performed under atmospheric, hyperoxic conditions, yet accumulating evidence confirms that cellular responses and proteome profiles differ significantly in cells under physiological O_2_ levels mimicking *in vivo* normoxia [1,4,11,39,74]. We have previously established that adaptation of EC to physiological O_2_ levels alters agonist-induced Ca^2+^ signaling, NO bioavailability, Fe^2+^ oxidation and NRF2-regulated gene expression [1,4,6,11,74] and now report that physioxia modulates ion channel activity. Although treatment of HUVEC and hCMEC/D3 cells with channel inhibitors confirmed the contribution of BK, IK and SK channels, a remaining current was recorded in HUVEC (36–47 %) and in hCMEC/D3 (18–56 %) under 18 kPa and 5 kPa O_2_. This remaining current may reflect the presence of other conductances reported in both cell types, such as non-selective PIEZO and TRP4 channels or CNG channels [36,38,[61], [62], [63]], which we have not further investigated. However, immunoblotting and immunocytochemical studies confirm that HUVEC and hCMEC/D3 cells express SK3 (SK), SK4 (IK) and KCa1.1 (BK) channels, and expression was unaffected by changes in pericellular O_2_.

### 4.1 Effects on BK channels

The partial TEA block of whole-cell currents further supports evidence for an enhanced activation of BK currents under physioxia (Fig. 4, Fig. 5; 38 % *versus* 21 % block in HUVEC; 42 % *versus* 26 % block in hCMEC/D3). BK channels are directly modulated by NO *via S*-nitrosylation of cysteine-thiol residues of the channel protein [23,49,75]. Interestingly, SNP and NOCys increase channel open probability which is also observed following incubation with dithiothreitol (DTT) and glutathione [57]. We and others have previously reported that under hyperoxic conditions (18 kPa), NO can potentiate whole-cell outward (including Ca^2+^-activated) currents in HUVEC [25] and in rabbit aortic smooth muscle cells [49]. In HUVEC, NO increases BK channel currents and its open probability [49] by different mechanisms: i) direct action on the channel proteins (*via S*-nitrosylation) or ii) indirect action *via* cGMP-mediated phosphorylation [31,69], with some mechanisms of regulation targeting different channel subunits. The complexity of subunit interactions, and the fact that α- and β-subunits have cysteine-rich segments [[76], [77], [78]], provides the basis for redox modulation of this channel [79] which may underlie the differential BK current contributions in HUVEC and hCMEC/D3 cells adapted to 18 kPa or 5 kPa O_2_.

### 4.2 Effects on SK and IK channels

Our data in both HUVEC and hCMEC/D3 further show a contribution of IK and SK channels to the overall whole-cell current based on the partial whole-cell current sensitivities to TRAM-34 and apamin, respectively. Addition of apamin and TRAM-34, in the presence of TEA, did not reduce currents further under hyperoxic conditions (Figs. 4B and 5B). However, when assessing currents in hCMEC/D3 under 5 kPa O_2_, we observed larger contributions of TRAM-34- and apamin-sensitive currents confirming significant contributions of IK and SK currents (∼21 % and ∼19 %, respectively).

This observation can be explained by different signaling effects. Firstly, any increase in intracellular Ca^2+^ would potentiate any of the three Ca^2+^-dependent K^+^ currents. However, in particular in HUVEC, there is no evidence that basal intracellular Ca^2+^ levels are increased under 5 kPa O_2_ adaptations [11]. A second explanation could relate to changes in redox and associated activation of currents in low redox environments. Redox stress has been shown to suppress SK channels [51] and low redox levels and antioxidants favour activation of SK channels [80]. The observed increases in SK contributions in the present study to the overall current could thus reflect redox-sensitive regulation.

A further possible explanation for enhanced IK and SK currents under 5 kPa O_2_ relates to that fact that CaM can be regulated by phosphatase 2A (PP2A) and casein kinase II (CK2) through CaM phosphorylation and dephosphorylation, thereby altering the SK channel apparent Ca^2+^ sensitivity [81]. We have previously reported enhanced expression and membrane targeting of PP2A in HUVEC adapted to 5 kPa O_2_ [11], pontentially linking oxygen- and phosphatase-dependent modulation of SK and IK channels. Thus, lower redox distress, enhanced NO bioavailability [6,49,69] and phosphatase activity may be responsible for the augmented outward currents under physioxia in the absence of altered Ca^2+^-mediated channel activation [11]. Although fewer studies have investigated IK modulation directly, H_2_O_2_ and other thiol reactive agents such as 5,5′-dithio-bis-(2-nitrobenzoic acid) can inhibit IK channel activation in bovine aortic EC which is partly restored by the disulfide reducing agent DTT or reduced glutathione [82].

### 4.3 Effects on K_IR_ channels

Our data also suggest effects on inward rectifier channel activities in HUVEC adapted to 5 kPa O_2_. These currents show smaller amplitudes at 5 kPa and an NO potentiation only at 18 kPa O_2_. Redox mechanisms regulate such inward ATP-sensitive K^+^ channels. Reducing O_2_ levels in culture diminishes ATP levels [83] leading to subsequent activation of K_ATP_/Kir6.1 channels by low levels of ATP, whereas direct *S*-nitrosylation of Kir6.1/SUR1 inhibits ATP binding, thus preventing channel inactivation [23]. Our data in HUVEC adapted to 5 kPa O_2_ (Fig. 1A and B) are consistent with reports of Kir6.2 channel activation by NO/cGMP signaling [68]. Based on similar Kir6.1 expression in HUVEC under 18 kPa and 5 kPa O_2_ (Suppl Fig. 1), we propose that redox and/or ATP-mediated regulation might be responsible for the observed current changes. So far there are no other reports showing K_IR_ expression or function in hCMEC/D3. In reducing environments, recapitulating conditions in the cell cytosol of HUVEC adapted to 5 kPa O_2_, purified K_IR_ (Kir3.2) channels exhibit low basal activity. However, under oxidizing conditions, these K^+^ channels exhibit a high basal activity in the absence of agonists, consistent with our finding of enhanced K_IR_ activity in HUVEC under 18 kPa O_2_ (Fig. 1B).

Together, our data establish for the first time that adaptation of EC to physiological O_2_ levels, in absence of HIF-1α stabilization [4,11], results in altered electrophysiological phenotypes. Macro- and microvascular EC exhibit distinct differences in whole-cell currents under both hyperoxia and physioxia conditions, suggesting that long-term adaptation to lower O_2_, reflecting *in vivo*-like redox levels, modulates ion channel function. Whether the mechanisms are the consequence of direct redox modifications of the channel proteins or of secondary regulatory mechanisms remains to be determined.

## 5 Conclusions

Our findings suggest that regulation of K^+^ channels in endothelial cells under physiological O_2_ has profound and different effects compared to hyperoxia under traditional cell culture conditions. To our knowledge, these experiments provide the first comparative profiling of ion channel activity in HUVEC and hCMEC/D3 adapted long-term to physioxia (5 kPa O_2_). Our findings show that ambient oxygen levels critically affect K^+^ channel activities and membrane potentials in endothelial cells *in vitro*, with insights for studies of endothelial function, vascular tone and the design and use of experimental models *in vitro* for high throughput drug discovery and clinical translation.

The present study has implications beyond regulation of vascular tone. Leukocyte transmigration in postcapillary venules depends on endothelial barrier integrity, NO signaling and K^+^ channel activity [[84], [85], [86]]. Enhanced NO bioavailability and altered K^+^ channel function under physiological O_2_ may influence junctional dynamics and paracellular permeability, potentially modulating immune cell trafficking. Future studies employing transmigration assays should investigate whether physioxia-induced changes in endothelial electrophysiology affect leukocyte adhesion and transmigration.

## CRediT authorship contribution statement

**Fan Yang:** Data curation, Formal analysis, Investigation, Methodology, Writing – original draft, Writing – review & editing. **Ashia Wheeler-Crawford:** Investigation. **Alan McIntyre:** Methodology. **Giovanni E. Mann:** Conceptualization, Funding acquisition, Methodology, Project administration, Resources, Supervision, Visualization, Writing – original draft, Writing – review & editing. **Joern R. Steinert:** Conceptualization, Data curation, Formal analysis, Funding acquisition, Investigation, Project administration, Supervision, Writing – original draft, Writing – review & editing.

## Declaration of competing interest

The authors declare not competing interests.

## Data Availability

Data will be made available on request.
