# Supplementary material for: Physiological oxygen levels reset K+ channel activity in human vascular endothelial cells
Source: Redox Biol. 2025 Dec 18;89:103981. doi: 10.1016/j.redox.2025.103981 (PMC12808508; doi:10.1016/j.redox.2025.103981)
Supplement: Multimedia component 1 [file mmc1.docx]

**Supplementary Information**

**Physiological oxygen levels reset K^+^ channel activity in human vascular endothelial cells**

Fan Yang, Ashia Wheeler-Crawford, Alan McIntyre, Giovanni E. Mann^*^ and Joern R. Steinert^*^

Figures:

HUVEC


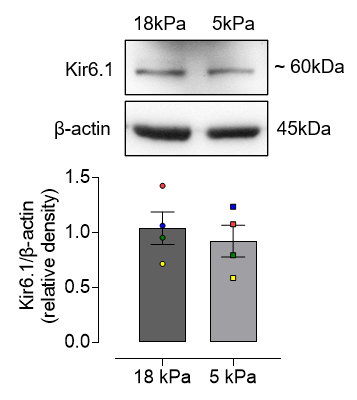


**Suppl Figure 1. Expression of KCNJ8 in human umbilical vein endothelial cells adapted to 5 kPa versus 18 kPa O_2_**.

Immunoblot analysis of KCNJ8 (Kir6.1, K_ATP_) channel expression in human umbilical vein endothelial cells (HUVEC) adapted for 5 days to 5 kPa or 18 kPa O_2_ reveals no difference (ratio channel protein/ß-actin, HUVEC: 5 kPa: 0.92±0.14, 18 kPa: 1.04±0.14 (*P*=0.59); n=3 independent cultures, Student’s *t*-test). Data denote mean ± S.E.M.

Original complete Western blot gels:

**HUVEC**

KCNJ8 (Kir6.1) Cultures 1-4


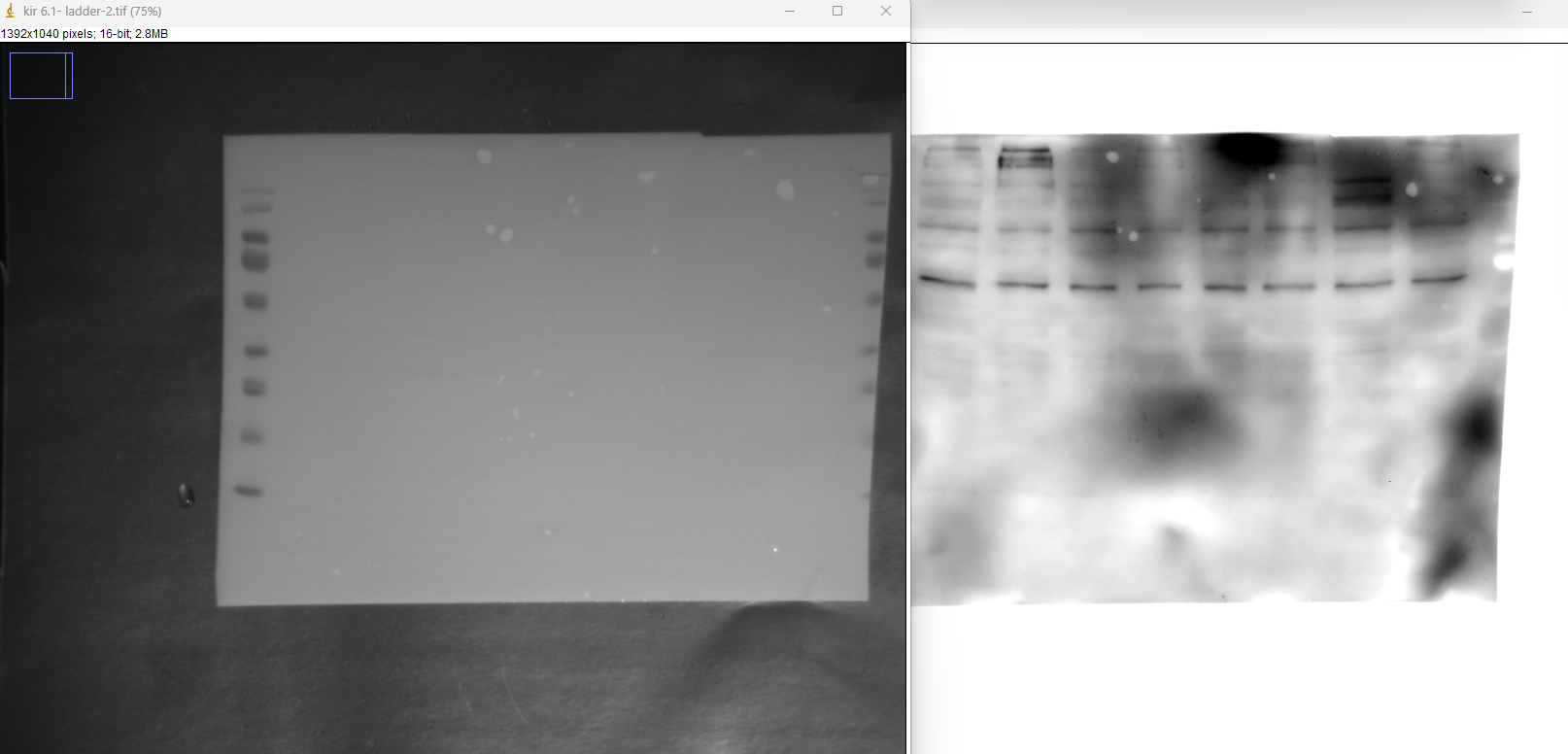


18 5 18 5 18 5 18 5kPa O_2_

KCa2.3 Cultures 1-4


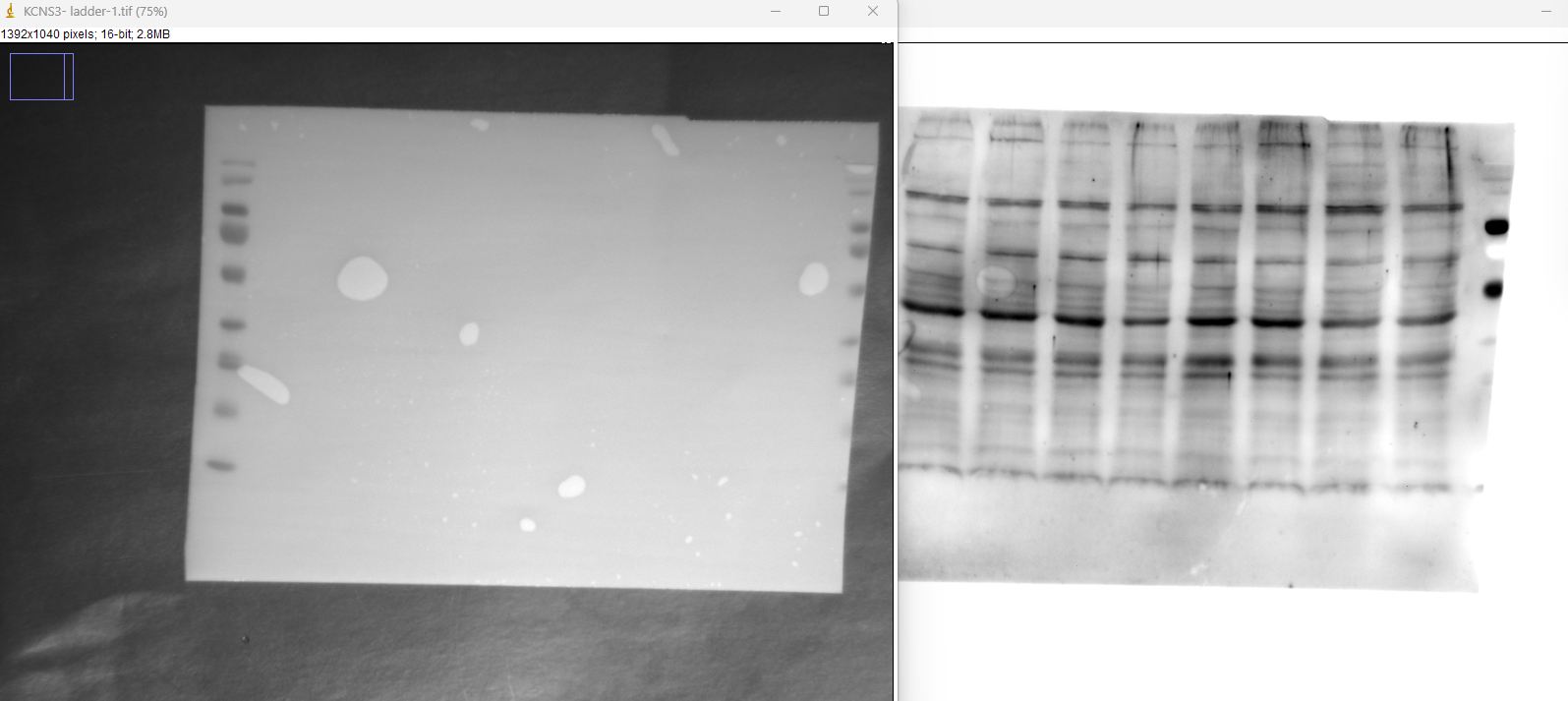


18 5 18 5 18 5 18 5kPa O_2_

β-actin


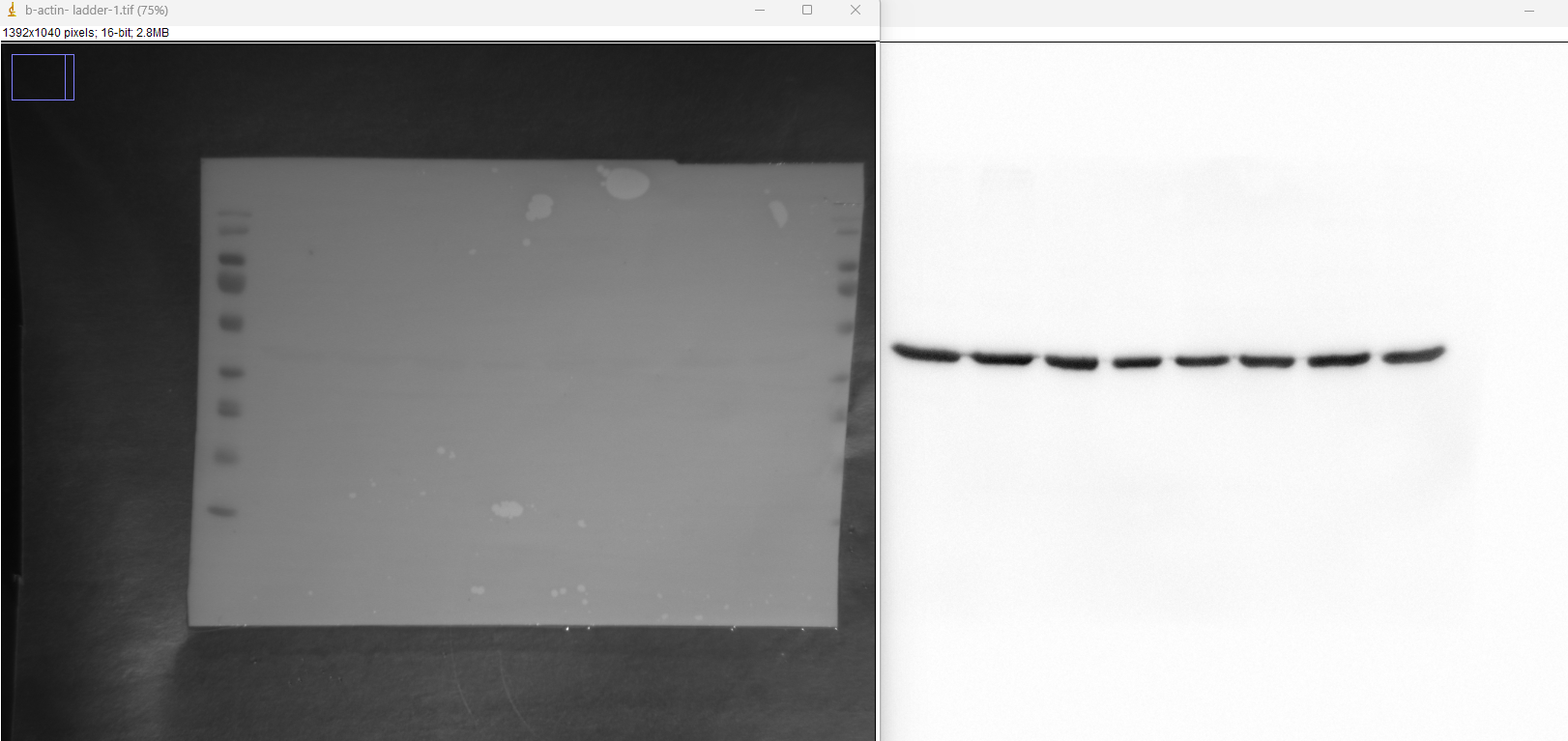


18 5 18 5 18 5 18 5kPa O_2_


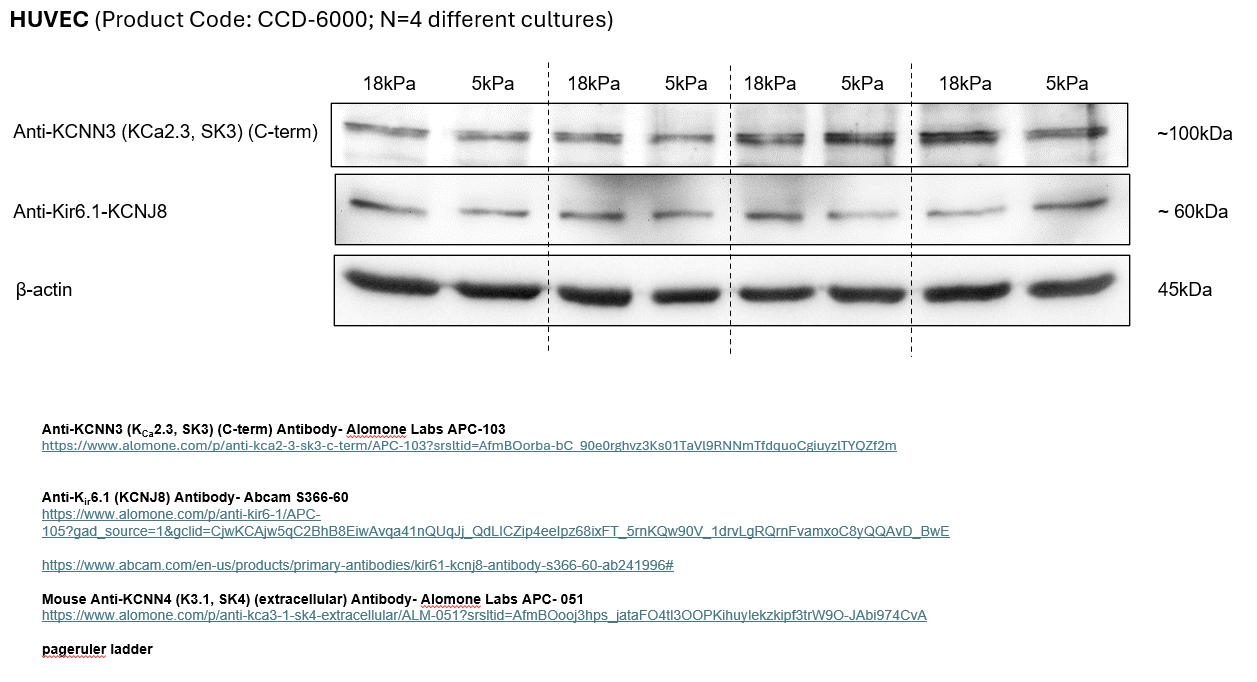


**Supp Fig 2A:** **Full gels of HUVEC WB shown in Fig 6.** Experiments were run in 4 independent cultures, lanes of immunostained proteins used for analysis are highlighted in dark dotted box with examples extracted for Fig 6 shown in red dotted box.

**hCMEC/D3 cells**

KCa2.3 Culture 1-3


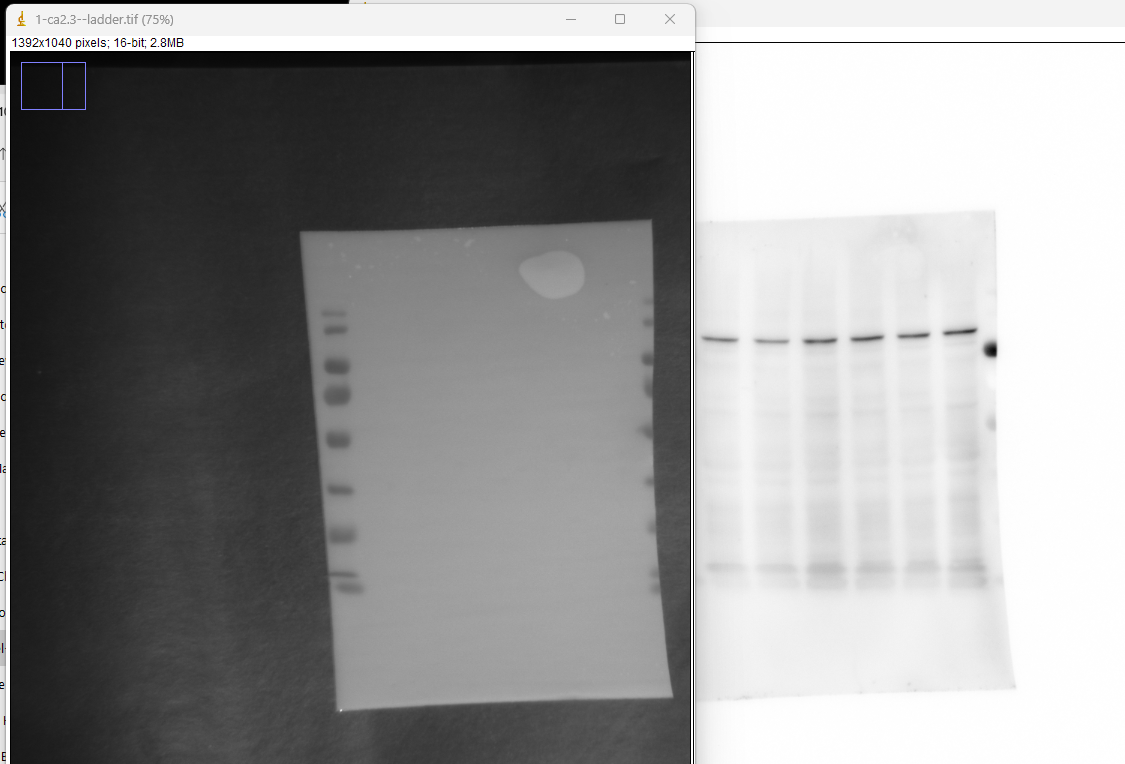


18 5 18 5 18 5kPa O_2_

β-actin


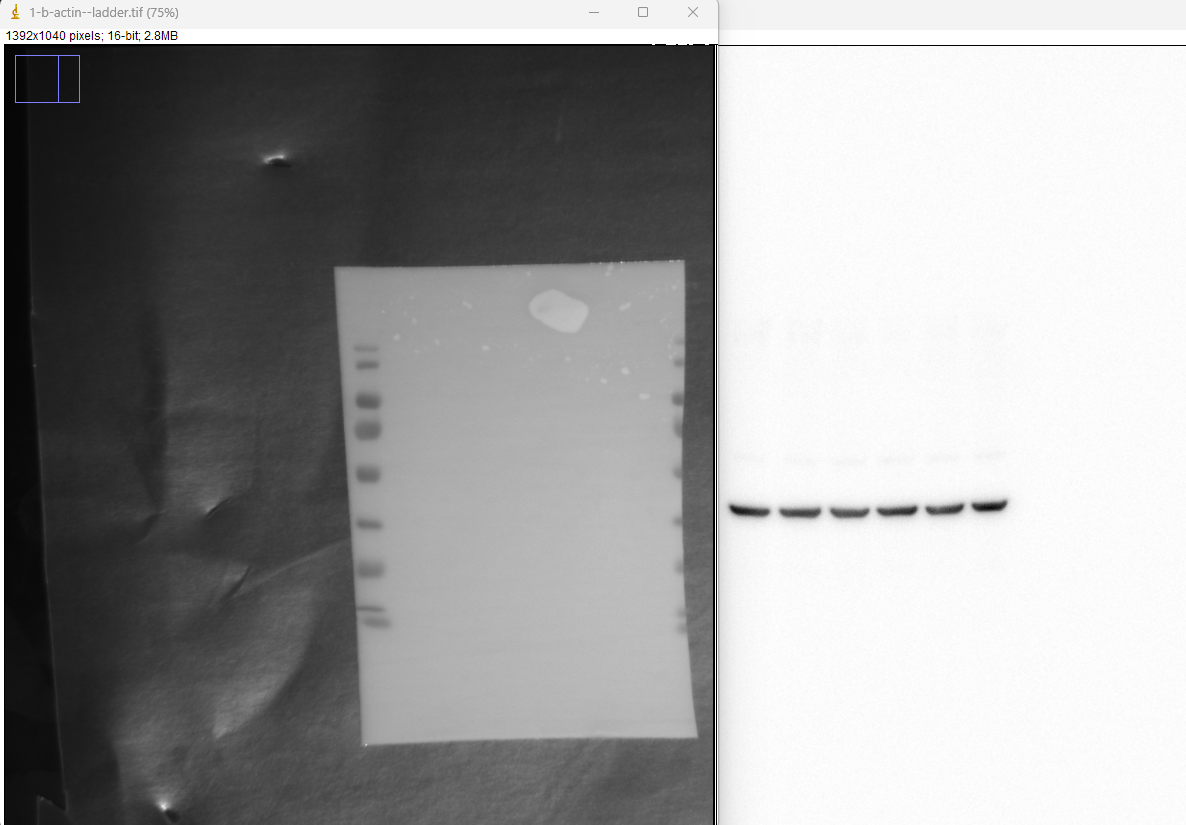


18 5 18 5 18 5kPa O_2_


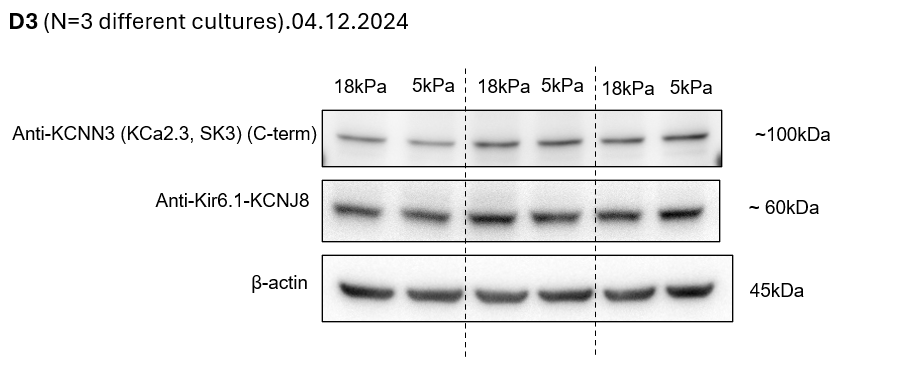


**Supp Fig 2B:** **Full gels of hCMEC/D3 cell WB shown in Fig 6.** Experiments were run in 3 independent cultures, lanes of immunostained proteins used for analysis are highlighted in dark dotted box with examples extracted for Fig 6 shown in red dotted box.
